# Protective mechanisms of exogenous melatonin on chlorophyll metabolism and photosynthesis in tomato seedlings under heat stress

**DOI:** 10.3389/fpls.2025.1519950

**Published:** 2025-02-04

**Authors:** Wangwang An, Guangzheng Wang, Jianhua Dou, Yonghai Zhang, Qing Yang, Yongmei He, Zhongqi Tang, Jihua Yu

**Affiliations:** ^1^ College of Horticulture, Gansu Agricultural University, Lanzhou, China; ^2^ State Key Laboratory of Aridland Crop Science (Gansu Agricultural University), Lanzhou, China

**Keywords:** tomato, melatonin, high temperature, photosynthesis, chlorophyll metabolism

## Abstract

Elevated temperatures severely affect plant growth, reducing yield and quality. Melatonin (MT), a plant biomolecule, is known to enhance stress tolerance, but its role in heat resistance and underlying mechanisms require further exploration. This study investigates MT’s regulatory effects on chlorophyll metabolism and photosynthesis in tomato seedlings under high-temperature stress (40°C). Tomato seedlings treated with 100 μmol MT showed improved physiological and photosynthetic performance under heat stress. MT application increased osmolytes (proline and soluble sugar), enhanced antioxidant enzyme activities [catalase (CAT), peroxidase (POD), ascorbate peroxidase (APX)], and reduced oxidative damage markers (H_2_O_2_, O_2_^−^, malondialdehyde, and conductivity). Photosynthetic parameters, including key enzyme activities [sedoheptulose-1,7-bisphosphatase (SBPase), ribulose-1,5-bisphosphate carboxylase/oxygenase (Rubisco), and NADP-dependent glyceraldehyde-3-phosphate dehydrogenase (NADP-GAPDH)], photochemical efficiency [Fv/Fm and Y(II)], and photochemical quenching (Qp), were significantly improved, restoring the OJIP curve and enhancing photosynthesis. MT also regulated chlorophyll metabolism by promoting synthesis [increasing chlorophyll *a* and *b*, 5-aminolevulinic acid (ALA), Mg-protoporphyrin (Mg Proto), and protochlorophyllide (Pchlide) levels] and upregulating synthesis genes (*SlHEMA1*, *SlPORB*, *SlPORC*, and *SlCHLI*) while inhibiting degradation genes (*SlCLH1*, *SlCLH2*, *SlPAO*, *SlPPH*, and *SlRCCR*). These findings demonstrate that MT enhances tomato heat tolerance by protecting chlorophyll metabolism and photosynthesis, offering a theoretical basis for improving crop resilience to heat stress.

## 1 Introduction

Tomatoes (*Solanum lycopersicum* L.) are one of the most important vegetable crops globally and hold a prominent place in China’s greenhouse vegetable industry (Razifard et al., 2020). Tomatoes are highly sensitive to ambient temperature, as thermophilic plants, with both excessively high and low temperatures impacting their growth and development (Adams et al., 2001). Temperatures exceeding 35°C negatively impact tomato growth (Hatfield and Prueger, 2015). High-temperature stress can cause leaf yellowing, stunted growth, and tissue wilting in tomatoes, significantly reducing both yield and quality (Aldubai et al., 2022). High-temperature stress induces various types of physiological damage in tomato seedlings. High temperature can severely impair cell membranes, compromising their selective permeability and leading to electrolyte leakage and increased conductivity. Excessive reactive oxygen species (ROS) interact with unsaturated fatty acids, exacerbating cellular damage (Yang et al., 2019). Furthermore, high temperatures seriously affect chlorophyll metabolism in tomato seedlings, thereby inhibiting photosynthesis (Ding et al., 2017). High temperatures compromise membrane integrity, accelerating chlorophyll degradation and causing chlorosis in leaves. Enzymes in the chlorophyll degradation pathway, such as magnesium-free pheophorbide a oxygenase (PAO), pheophytin pheophorbide hydrolase (PPH), chlorophyllase (CLH), and red chlorophyll catabolite reductase (RCCR), play essential roles (Jahan et al., 2022). Research indicates that high-temperature stress significantly reduces chlorophyll content in *Agrostis* sp., primarily due to the upregulation of the *PPH* and *CLH* genes and increased PPH activity, which accelerates chlorophyll degradation (Jespersen et al., 2016). ABI5 promotes chlorophyll degradation under heat stress by regulating MYB44 stability in cucumber. ABI5 directly binds to the promoters of the PPH and PAO genes, enhancing their expression and thus accelerating chlorophyll degradation (Liu et al., 2023). Elevated temperatures and salt stress significantly enhance PAO and RCCR transcription in both cucumbers and peppers (Xiao et al., 2015; Zhou et al., 2016). Maintaining chlorophyll homeostasis and enhancing photosynthesis in tomato seedlings under high-temperature stress are crucial for improving heat tolerance. Moreover, abscisic acid, auxin, and ethylene, as plant hormones, are among the essential compounds for mitigating heat stress (Verma et al., 2016). Particularly, the periodic changes at physiological, biochemical, and molecular levels under high-temperature stress are often closely associated with the tight regulation of these hormones (Peer et al., 2020). Under high-temperature stress, the internal abscisic acid (ABA) levels in plants significantly increase, reducing water loss and oxidative damage by regulating stomatal closure and the activity of antioxidant enzymes. Specifically, high temperatures promote the synthesis and accumulation of ABA in leaves, activate ABA signaling pathways, and regulate the degree of stomatal closure, thereby enhancing leaf transpiration. Additionally, ABA can induce the expression of a series of stress-related genes, such as heat shock protein (HSP) genes, which protect cellular protein structures and prevent protein denaturation and functional loss caused by high temperatures (Zou et al., 2009; Joshi and Chinnusamy, 2014). Auxin is a key regulatory factor in plant growth, and its levels and distribution patterns undergo significant changes under high-temperature stress. Studies have shown that under high-temperature stress, the contents of indole-3-acetic acid (IAA) and gibberellins (GAs) in rice decrease significantly, while ABA levels increase markedly. Moreover, the longer the stress duration, the more pronounced the reduction in IAA and GAs (Tang et al., 2008). Ethylene, as a gaseous signaling molecule, plays a vital role in plant responses to stress conditions (Tao et al., 2015). Exogenous ethylene can activate various stress-responsive proteins to enhance plant thermotolerance. Ethylene signaling also contributes to alleviating heat-induced stress in plants by reducing oxidative stress and maintaining chlorophyll content, thereby improving heat resistance (Gautam et al., 2022).

Melatonin (*N*-acetyl-5-methoxytryptamine) is an effective free radical scavenger and antioxidant that supports plant growth and resistance to abiotic stress, reducing stress-induced damage in plants (Cen et al., 2020). Studies have shown that melatonin (MT) can directly scavenge excessive ROS under stress and enhance antioxidant enzyme activity, protecting plants from oxidative stress (Sharma and Zheng, 2019). MT is an effective antioxidant, crucial for enhancing antioxidant enzyme activity and maintaining ROS balance (Tan et al., 2000). MT applications improve mineral absorption and utilization, fostering plant growth, germination, root development, and photosynthesis (Hassan et al., 2022). MT significantly contributes to the improvement of plant resistance to elevated temperatures. Spraying 100 μmol·L^−1^ MT mitigates heat-induced damage in strawberries by enhancing the antioxidant mechanism (Manafi et al., 2022). MT-treated rice exhibited enhanced antioxidant capacity, resulting in increased photosynthesis, greener leaves, elevated photosynthetic parameters (Fv/Fm and PI), and improved heat tolerance (Fan et al., 2022). MT application preserves chloroplast integrity in chrysanthemums, decreases K and J points in the OJIP curve, and mitigates high-temperature inhibition on photosynthesis (Qi et al., 2021). Photosynthesis is a crucial determinant of plant growth and development. Studies have shown that high temperatures reduce the photosynthetic rate; however, MT application downregulates chlorophyll degradation genes in Chinese cabbage, delays senescence, and protects photosynthesis (Tan et al., 2019). To date, various plant hormones and signaling molecules have been found to be involved in plant responses to heat stress (Hasanuzzaman et al., 2013). Exogenous melatonin can increase the levels of endogenous melatonin and cytokinins in ryegrass under high-temperature stress while reducing the content of abscisic acid (Zhang et al., 2017).

Numerous studies have shown that MT effectively alleviates plant stress; however, research on MT’s role in enhancing tomato heat tolerance is limited. Additionally, no studies have explored MT’s potential to protect chlorophyll metabolism and mitigate high-temperature effects on tomato photosynthesis, thus enhancing heat tolerance. This research gap remains unexplored. Therefore, this study investigated the effects of exogenous melatonin on tomato seedling growth under high-temperature stress to elucidate melatonin-induced heat resistance mechanisms at the physiological level. This study aims to provide a theoretical foundation for applying melatonin in research on tomato stress resistance.

## 2 Materials and methods

### 2.1 Plant materials and experimental design

This study used the tomato cultivar ‘CR’ (*S. lycopersicum* cv. Condine Red) as experimental material, which was provided by the Department of Horticulture, School of Agriculture and Biotechnology, Zhejiang University. The seeds were soaked in water at 50°C–55°C for 10 hours, then evenly scattered on a 1-cm-thick layer of vermiculite, covered with an additional 0.5 cm of vermiculite, and thoroughly watered to promote germination. After germination and full expansion of the two cotyledons, the seedlings were transplanted into nutrient soil (substrate:vermiculite:perlite = 3:2:1). The tomatoes were cultivated and managed in a climate chamber (JNR-518C-3, Ningbo Jiangnan Instrument Factory, Zhejiang, China) at 18°C/28°C, 60% humidity, 12 hours of light/12 hours of darkness, and a light intensity of 350 μmol·m^−2^·s^−1^. Experimental treatments began when the tomatoes had developed five leaves and one heart.

When tomato seedlings had developed five leaves and one heart, melatonin (purchased from Beijing Solarbio Biotechnology Co., Ltd., Beijing, China) was administered at 100 μmol·L^−1^ each night at 9:00 p.m. After a 3-day pretreatment, seedlings underwent 3 days of continuous high-temperature treatment, during which samples were collected. Four treatments were tested, each with three replicates of 100 plants, totaling 400 seedlings. The control group was maintained in a climate chamber at 28°C/18°C, with 60% relative humidity and a light intensity of 350 μmol·m^−2^·s^−1^. High-temperature-treated seedlings were placed in a climatic chamber at 40°C, with 60% relative humidity and a light intensity of 350 μmol·m^−2^·s^−1^. The experimental design is shown in **Table 1**.

**Table 1:** Experimental design.

Processing name	Treatment
CK	Seedlings were sprayed with deionized water at room temperature of 28°C/18°C
MT	Spray 100 μmol·L^−1^ melatonin on the leaves of seedlings at room temperature of 28°C/18°C
HT	Spray deionized water on seedlings at a high temperature of 40°C
HT+MT	Spray 100 μmol·L^−1^ melatonin on the leaves of seedlings at a high temperature of 40°C

CK, control; MT, melatonin; HT, high temperature.

### 2.2 Measurement of plant growth indicators

Three seedlings from each treatment group were randomly selected for height and stem thickness measurements using a straightedge and vernier caliper. The washed plants were drained of surface water, and their fresh weight was measured. For dry weight determination using the constant weight drying method, fresh leaves were placed in an oven at 105°C for 30 minutes to deactivate green tissue and then dried at 80°C until a constant weight was achieved.

### 2.3 Measurement of cell membrane permeability, malondialdehyde, soluble sugar, and proline contents

Conductivity was measured using the method of Wang et al. Tomato leaves weighing 0.2 g were placed in a test tube. Subsequently, 20 mL of deionized water was added to the test tube, and the mixture was shaken for 1 hour at a temperature of 28°C. After this period, the initial conductivity was measured, denoted as E1. Next, the tomato leaves were subjected to a boiling water bath at 95°C for a duration of 20 minutes. Once the heating process was complete and the sample was cooled, the conductivity was measured again, referred to as E2. Finally, the final conductivity was calculated using the following formula: (E2/E1) × 100% (Wang F. et al., 2016).

Malondialdehyde content was determined using the thiobarbituric acid method (Chamseddine et al., 2009). To begin the experiment, 0.5 g of tomato leaves was placed into a test tube. Subsequently, 10 mL of phosphate solution was added along with a 10% trichloroacetic acid (TCA) solution. The mixture was incubated in a water bath at 30°C for a duration of 30 minutes. Following this, the mixture was allowed to cool and then centrifuged to separate the components. The supernatant was carefully extracted and combined with an equal volume of thiobarbituric acid (TBA). The resulting solution was heated in a water bath at 100°C for an additional 30 minutes. After being cooled, the absorbance of the solution was measured at a wavelength of 532 nm.

Soluble sugar content was measured using the anthrone method: 0.1 g of tomato leaves was added with 10 mL of an 80% ethanol solution, ground, mixed, and shaken for 24 hours. Following centrifugation, 0.5 mL of the supernatant was extracted and combined with 2.5 mL of anthrone solution. The mixture was then heated in a water bath at 40°C for a duration of 30 minutes. After allowing the solution to cool, the absorbance was measured at a wavelength of 625 nm (Das et al., 2024).

The content of proline (Pro) content in the leaves was determined using the ninhydrin method (Hayat et al., 2008). The tomato sample weighing 0.2 g was added with 5 mL of a 3% sulfosalicylic acid, 2 mL of ninhydrin, and 2 mL of acetic acid. It was subjected to a boiling water bath for 15 minutes. Following centrifugation, the supernatant was collected. The absorbance was measured at a wavelength of 520 nm.

### 2.4 Measurement of reactive oxygen species, antioxidant enzyme activity, photosynthetic enzyme activity, and tissue staining

To begin the experiment, 0.1 g of tomato leaves was added with an appropriate extraction solution. The mixture was thoroughly homogenized in an ice bath. Subsequently, the homogenate was centrifuged at 12,000 *g* and 4°C for a duration of 20 minutes. After centrifugation, the supernatant was carefully aspirated, and the concentration of superoxide anion was quantified following the instructions provided in the Solarbio Biological Kit (Beijing, China). In a separate procedure, 0.1 g of tomato leaves was added to the extraction solution and homogenized in an ice bath. The homogenate was centrifuged at 8,000 rpm and 4°C for 20 minutes. Again, the supernatant was aspirated, and the hydrogen peroxide content was measured in accordance with the guidelines outlined in the Solarbio Biological Kit (Beijing, China).

Tomato leaves weighing 0.1 g were added with an appropriate extraction solution. Subsequently, the mixture was thoroughly ground while maintaining an ice bath to prevent thermal degradation. Following this, the homogenate was centrifuged at a force of 8,000 rpm and a temperature of 4°C for a duration of 10 minutes. The supernatant was aspirated, and the enzyme activities of superoxide dismutase (SOD), peroxidase (POD), catalase (CAT), and ascorbate peroxidase (APX) were assessed according to the Solarbio Biological Kit instructions (Beijing, China).

The enzyme activities of sedoheptulose-1,7-bisphosphatase (SBPase), ribulose-1,5-bisphosphate carboxylase/oxygenase (Rubisco), and NADP-dependent glyceraldehyde-3-phosphate dehydrogenase (NADP-GAPDH) were determined using the respective ELISA detection kits: the SBPase ELISA detection kit (Shanghai Youxuan Biotechnology, Shanghai, China; YX-22912P), the Rubisco ELISA detection kit (Shanghai Youxuan Biotechnology, YX-22015P), and the NADP-GAPDH ELISA detection kit (Shanghai Youxuan Biotechnology, YX-22917P).

Histostaining of H_2_O_2_ and O_2_^−^ was conducted using the method of Thordal-Christensen et al (Thordal Christensen et al., 1997). Tomato leaves were immersed in buffer solutions of 1 mg/mL diaminobenzidine staining method (DAB) and 0.5 mg/mL nitroblue tetrazolium staining method (NBT) for 1 hour and then cultured in the dark for 6 hours. Then, the tomato leaves were placed in a lactic acid/glycerol/ethanol decolorizing solution and boiled for decolorization to observe brown marks and blue marks.

### 2.5 Measurement of chlorophyll fluorescence parameters

The initial fluorescence (Fo), maximum fluorescence (Fm), maximum fluorescence yield (Fm′), initial fluorescence under light (Fo′), and steady-state fluorescence (Fs) of tomato leaves after 30 minutes of dark treatment were measured using an IMAP ING-PAM modulated fluorescence analyzer (Walz, Effeltrich, Germany). Then, according to the appropriate formulas, the maximum photochemical efficiency (Fv/Fm), the actual photochemical efficiency [Y(II)], the photochemical quenching coefficient (qP), and the non-photochemical quenching coefficient (NPQ) were computed (Lu et al., 2019).


Fv/Fm=(Fm-Fo)/Fm

Y(II)=((Fm'-Fs)/Fm')

qP=(Fm'-Fs)/(Fm'-Fo')

NPQ=(Fm-Fm')/Fm'

### 2.6 Measurement of rapid chlorophyll fluorescence kinetics (OJIP curve) and JIP-test parameters

Plant Efficiency Analyzer Handy PEA (Hansha Scientific Instruments Ltd., Shandong, China) was employed to assess the rapid chlorophyll fluorescence induction parameters of tomato leaves. Before the determination, the tomato leaves were fully adapted to the dark for 30 minutes and then induced by 3,000 μmol·m^−2^·s^−1^ red light for 2 s. The fast chlorophyll fluorescence induction kinetics curve (OJIP fluorescence induction curve) was subsequently determined. Five tomato seedlings were assessed for each treatment. According to the measured chlorophyll fluorescence rapid induction kinetic curve, various fluorescence parameters of the OJIP-test were calculated and analyzed according to Strasser’s method, as presented in **Table 2** (Hwang and Choo, 2017).

**Table 2:** Formula for calculating dynamic parameters of rapid chlorophyll fluorescence.

Parameter and formula	Explanation of the parameters
Vj = (Fj − Fo)/(Fm − Fo)	Relative variable fluorescence at J step
ABS/CSm ≈ Fm	Light energy absorbed per unit area
TRO/CSm = φPO·(ABS/CSm)	Light energy captured per unit area
ETO/CSm = ΨEo·(ABS/CSm)	Light energy per area for electron transport
DIo/CSm = (ABS/CS) − (TRo/CS)	Thermal dissipation per unit area
ABS/RC = Mo·(1/Vj)·(1/φPo)	Light energy absorbed by unit reaction center
TRo/RC = Mo·(1 − Vj)	Energy captured by unit reaction center for QA reduction
ETo/RC = Mo·(1/Vj)·ψo	Energy captured by unit reaction center for electron transfer
DIo/RC = (ABS/RC) − (TRo/RC)	Energy dissipated by unit reaction center

### 2.7 Determination of chlorophyll content

Tomato leaves were chopped and extracted with ethanol in the dark for 48 hours. Absorbance was measured at 665 nm, 649 nm, and 470 nm using Anwar’s method (Debona et al., 2017).


$$\text{Chlorophyll a concentration }\left({\text{C}}_{\text{a}}\right)=13.95\times {\text{A}}_{665}-6.8\times {\text{A}}_{649}$$

$$\text{Chlorophyll b concentration }\left({\text{C}}_{\text{b}}\right)=24.96\times {\text{A}}_{649}-7.32\times {\text{A}}_{665}$$

$$\text{Chlorophyll a+b concentration }\left({\text{C}}_{\text{a}+\text{b}}\right)=18.16\times {\text{A}}_{469}+6.63\times {\text{A}}_{665}$$

$$\text{Carotenoid concentration}=\left(1,000{\text{A}}_{470}-2.05\times \text{Ca}-114.8\times \text{Cb}\right)/248$$

### 2.8 Measurement of 5-aminolevulinic acid as a chlorophyll synthesis precursor

The tomato leaves were ground in 6 mL of acetic acid buffer while maintained in an ice bath. After centrifugation at 10,000 rpm for 15 minutes, the supernatant was collected, and subsequently, four drops of ethyl acetoacetate were added. The mixture was heated in a water bath at 100°C for 10 minutes and cooled to room temperature. An equal volume of fresh Escher reagent for color development was added for 15 minutes, shaken, and allowed to stand for 10 minutes. The Optical Density (OD) value was measured at 554 nm (Zhang Z. et al., 2015).

### 2.9 Measurement of protoporphyrin IX, Mg-protoporphyrin IX, and protochlorophyllide contents

Fresh tomato leaves were collected and added with 25 mL of 80% alkaline acetone solution (80% acetone, 20% ammonia, 1% concentration). They were ground, extracted, and soaked in the dark until the tissue turned white. The absorbance of the supernatant was measured at 575 nm, 590 nm, and 628 nm to determine the concentrations of the three intermediate products (Liu et al., 2015).


$$\text{Proto IX }\left({\text{μmol}\cdot \text{g}}^{-1}\text{FW}\right)=\left(0.18016\times \text{A}575–0.04036\times \text{A}628–0.04515\times \text{A}590\right)\times \text{V}/\text{FW}$$

$$\text{Mg-Proto IX }\left({\text{μmol}\cdot \text{g}}^{-1}\text{FW}\right)=\left(0.06077\times \text{A}590–0.01937\times \text{A}575–0.003423\times \text{A}628\right)\times \text{V}/\text{FW}$$

$$\text{Pchlide }\left({\text{μmol}\cdot \text{g}}^{-1}\text{FW}\right)=\left(0.03563\times \text{A}628+0.007225\times \text{A}590–0.02955\times \text{A}575\right)\times \text{V}/\text{FW}$$

### 2.10 Determination of melatonin content

A 0.1-g sample of tomato leaves was collected and thoroughly ground in extraction solution on an ice bath. The homogenate was then centrifuged at 8,000 g for 10 minutes at 4°C. The supernatant was collected, and the melatonin content was determined using an ELISA detection kit (Shanghai Youxuan Biotechnology Co., Ltd., YX-132000P) according to the manufacturer’s instructions.

### 2.11 Chlorophyll-related gene expression analysis

Following a 24-hour processing period, tomato seedling leaves were collected and subsequently frozen in liquid nitrogen before being stored in an ultra-low temperature freezer for gene expression measurement. Quantitative real-time PCR was employed to extract total RNA from tomato seedling leaves using the RNAprep Pure Plant Plus Kit from Tiangen Biotechnology (Beijing, China). The specific extraction method and process were carried out according to the instructions, and the final assessment of RNA purity and concentration was performed to determine if it could be used for the next step of testing. cDNA synthesis was performed utilizing the FastKing One Step RT Kit from Tiangen Biotechnology. The specific system and operational procedures were meticulously followed in accordance with the provided instructions. Fluorescence quantitative polymerase chain reaction (qPCR) was conducted utilizing synthesized cDNA as template primers as detailed in **Supplementary Table S1**, RT-PCR amplification was carried out using the Tiangen SuperReal PreMix Plus (SYBR Green) kit, and the amplification system strictly followed the manufacturer’s product instructions. Each treatment was repeated three times, and quantitative analysis was performed using the CT value method (Livak and Schmittgen, 2001).

### 2.12 Statistical analysis

After data collection, SPSS 22.0 (IBM, Armonk, NY, USA) was used for variance analysis, and Duncan’s test separated the means at a significance level of 0.05. The results are presented as mean ± standard error and plotted using OriginPro 2020 (OriginLab Inc., Northampton, MA, USA).

## 3 Results and analysis

### 3.1 Effects of exogenous melatonin on growth of tomato seedlings under high-temperature stress

**Figure 1:**
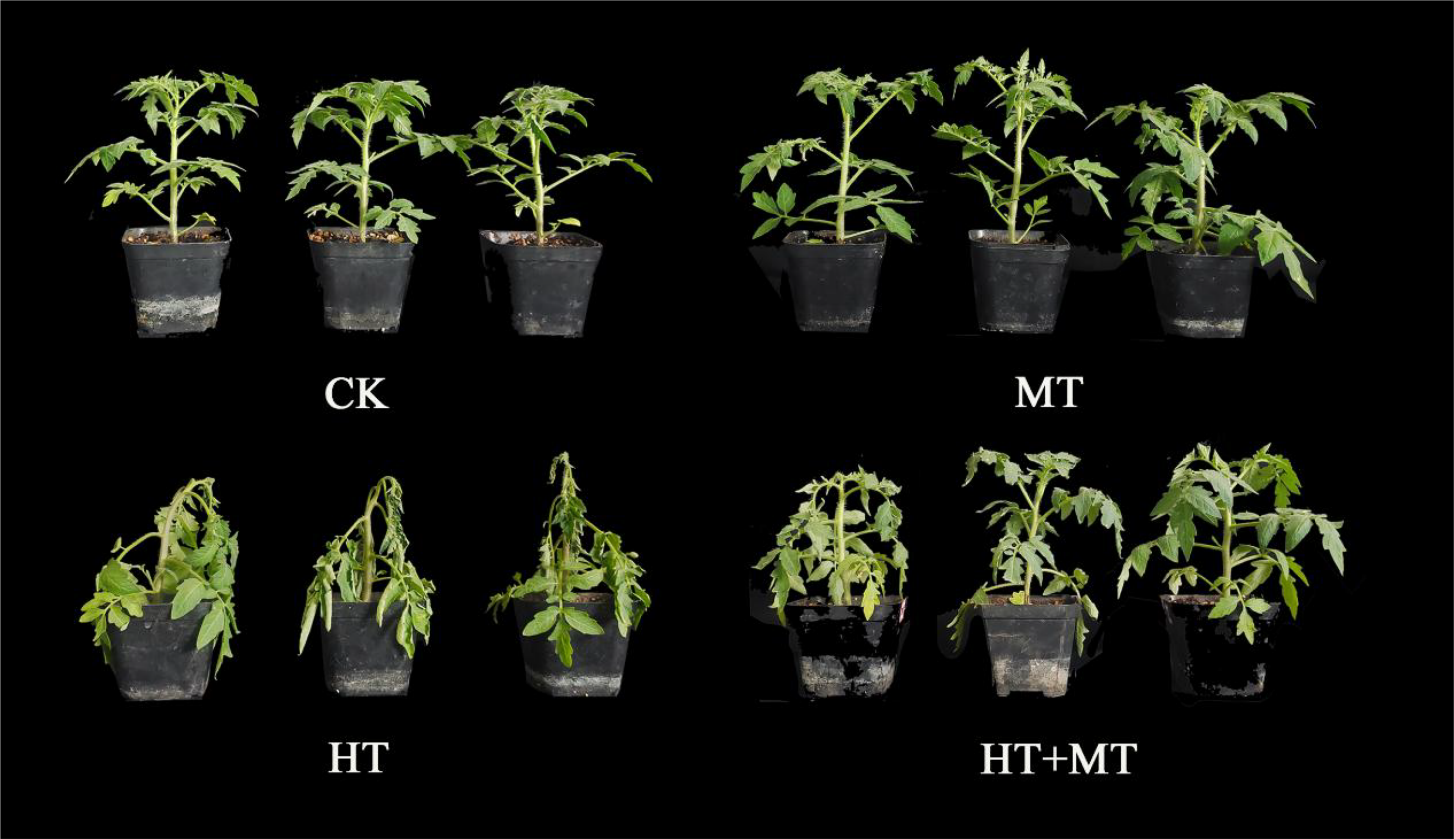
Phenotype of tomatoes treated with different methods.

Tomato seedlings sprayed with melatonin at room temperature showed no differences from control seedlings, indicating that MT application under these conditions does not induce stress in tomato seedlings. However, tomato seedlings exposed to high-temperature stress exhibited significant leaf wilting. Spraying with 100 μmol·L^−1^ MT alleviated leaf wilting symptoms under high-temperature stress (**Figure 1**; **Table 3**).

**Table 3:** Effects of exogenous melatonin on tomato growth under high-temperature stress.

Treatment	Apical new leaf	Lower old leaf
CK	Leaves smooth, dark green in color	Green color, flat leaves
MT	Leaves smooth, dark green in color	Green color, flat leaves
HT	Leaves curled, drooping	Leaves yellowing, wilting, drooping
HT+MT	Leaf edge slightly curled	Leaves slightly wilted, drooping

CK, control; MT, melatonin; HT, high temperature.

**Table 4** shows that high temperatures significantly impaired the growth of tomato seedlings. MT-treated tomato seedlings exhibited higher plant height, stem thickness, fresh weight, and dry weight than the control. High-temperature treatment significantly reduced plant height, stem thickness, above-ground fresh weight, above-ground dry weight, below-ground fresh weight, and below-ground dry weight by 13.36%, 13.50%, 46.77%, 37.33%, 42.85%, and 70.0%, respectively, compared to those in the control (CK). Under high-temperature treatment, MT-pretreated tomato seedlings showed improved growth. In the HT+MT treatment group, plant height, stem thickness, above-ground fresh weight, above-ground dry weight, below-ground fresh weight, and below-ground dry weight increased by 4.38%, 11.25%, 31.47%, 34.04%, 23.53%, and 1.00%, respectively, compared to those in the high-temperature (HT) group. This suggests that melatonin application effectively mitigates growth inhibition under high-temperature stress, although it does not fully restore growth to normal levels (**Table 4**).

**Table 4:** Effect of exogenous melatonin on tomato seedling biomass.

Treatment	Plant height/cm	Stem diameter/mm	Fresh shoot Weight/g	Fresh root Weight/g	Dry shoot Weight/g	Dry root Weight/g
CK	9.73 ± 0.15b	6.37 ± 0.08a	7.76 ± 0.3b	1.19 ± 0.91b	0.75 ± 0.53ab	0.1 ± 0.05b
MT	10.77 ± 0.26a	6.53 ± 0.17a	11.32 ± 0.56a	2.18 ± 1.58a	0.9 ± 0.71a	0.18 ± 0.05a
HT	8.43 ± 0.46c	5.51 ± 0.33b	4.13 ± 0.28d	0.68 ± 0.49c	0.47 ± 0.38c	0.03 ± 0.01c
HT+MT	8.8 ± 0.3bc	6.13 ± 0.22b	5.43 ± 0.27c	0.84 ± 0.69c	0.63 ± 0.38b	0.06 ± 0.03c

Different lowercase letters within the same column signify statistically significant differences between treatments (p < 0.05).

CK, control; MT, melatonin; HT, high temperature.

### 3.2 Role of exogenous melatonin in stabilizing cell membranes of tomato seedlings under high-temperature stress

**Figure 2:**
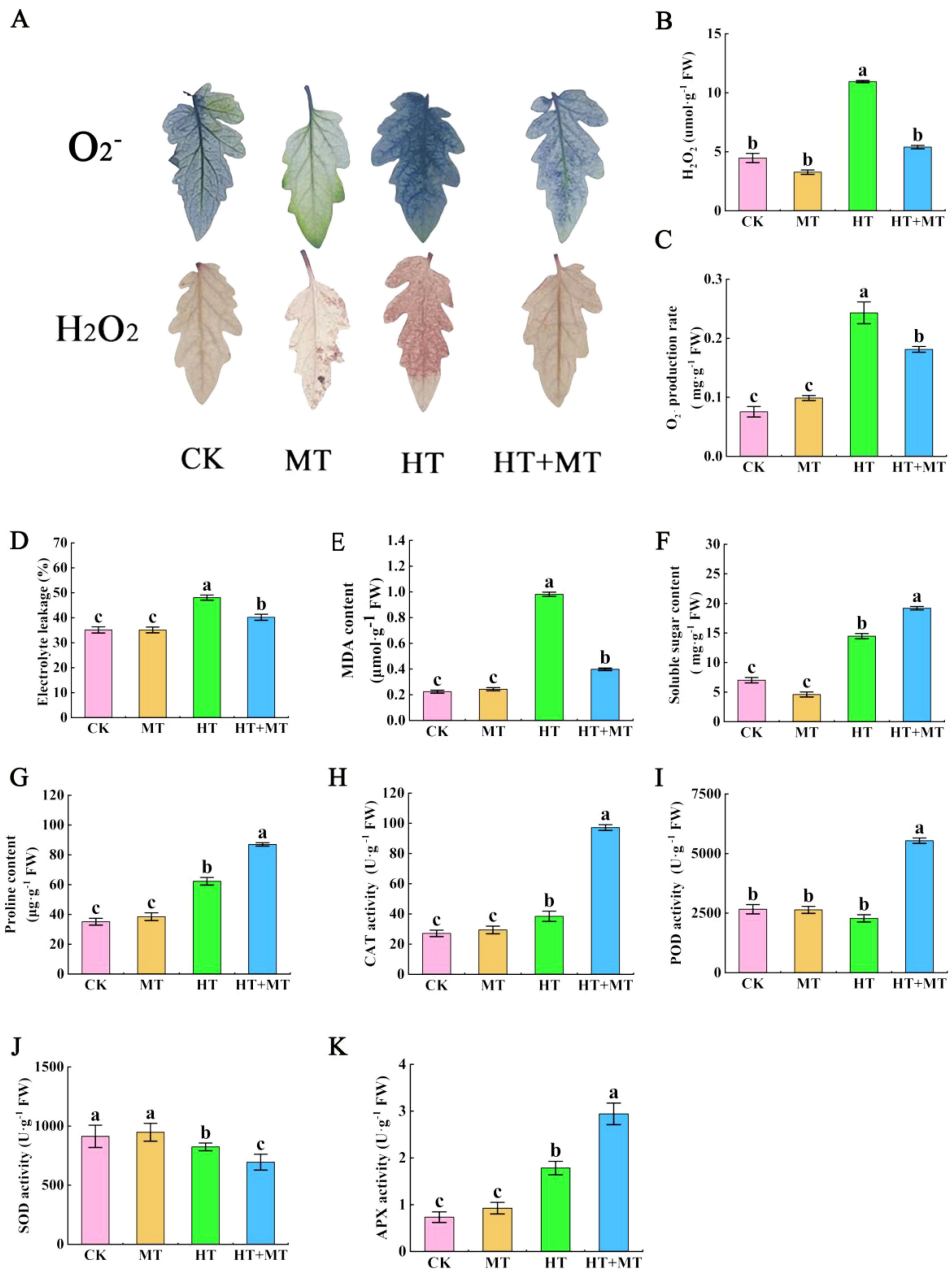
Effects of exogenous melatonin on the stability of tomato membrane system under high-temperature stress. **(A)** Tissue staining of reactive oxygen species. **(B)** H_2_O_2_ content. **(C)** O_2_^−^ content. **(D)** Electrolyte leakage. **(E)** Malondialdehyde (MDA) content. **(F)** Soluble sugar content. **(G)** Proline content. **(H)** Catalase (CAT) activity. **(I)** Peroxidase (POD) activity. **(J)** Superoxide dismutase (SOD) activity. **(K)** Ascorbate peroxidase (APX) activity. The results presented the means ± SE of three independent experiments. The different lowercase letters above the bars indicate significant differences between treatments according to Duncan’s test (p < 0.05).

The impact of melatonin on ROS in tomato seedling leaves under high-temperature stress was analyzed. In the HT treatment group, H_2_O_2_ and O_2_^−^ levels increased significantly by 144.95% and 221.79%, respectively, compared to those in the CK group. Conversely, the HT+MT treatment effectively controlled H_2_O_2_ and O_2_^−^ levels, reducing them by 50.68% and 25.49%, respectively, compared to those in the HT group (**Figures 2B**, **C**). Tissue staining results showed a similar trend. The results indicate that the application of MT plays a significant role in eliminating reactive oxygen species, thereby strengthening the antioxidant defense mechanism (**Figure 2A**).

The effect of exogenous melatonin on cell membrane permeability in tomato seedlings under high-temperature stress was analyzed. In the HT treatment group, relative conductivity in tomato leaves increased by 36.87%, and malondialdehyde (MDA) content rose by 337.54% compared to those in CK. In the HT+MT treatment group, relative conductivity and MDA content were significantly lower than those in the HT group, decreasing by 16.35% and 59.46%, respectively (**Figures 2D, E**). Under high-temperature stress, tomato seedlings accumulated osmotic regulators, such as proline and soluble sugars, to mitigate heat-induced damage. Compared to those in CK, proline content in the HT group increased by 77.19%, and soluble sugar content rose by 106.12%. Following the HT+MT treatment, proline and soluble sugar levels in tomato seedlings increased further by 39.73% and 32.74%, respectively, compared to those in the HT group (**Figures 2F, G**).

Under conditions of high-temperature stress, alterations in the activity of cellular antioxidant enzymes signify the plant’s capacity to tolerate heat stress. During these stressful conditions, the activity of antioxidant enzymes is heightened to eliminate surplus reactive oxygen species. Compared to CK, HT treatment significantly increased CAT and APX enzyme activities by 41.66% and 143.08%, respectively, while POD activity showed minimal change, and SOD activity significantly decreased. In the HT+MT group, CAT, POD, and APX activities further increased by 152.94%, 142.71%, and 108.02%, respectively, compared to those in the HT group, while SOD activity decreased (**Figures 2H–K**).

### 3.3 Influence of exogenous melatonin on photosynthetic characteristics and chlorophyll fluorescence kinetics in tomato seedlings under heat stress

**Figure 3:**
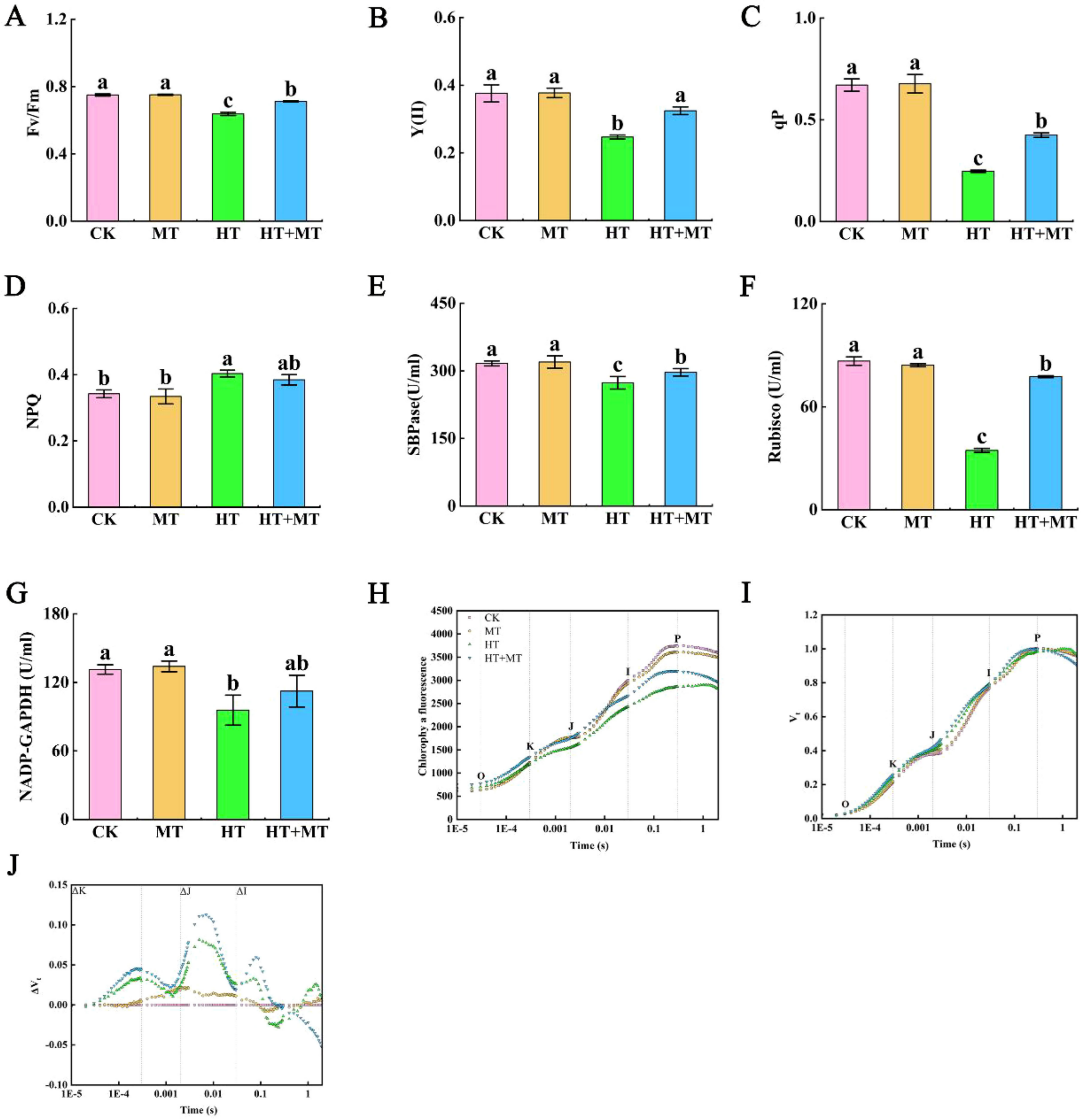
Effects of exogenous melatonin on photosynthetic system of tomato seedlings under high-temperature stress. **(A)** Maximum photochemical efficiency (Fv/Fm). **(B)** Actual photochemical efficiency, denoted as Y(II). **(C)** Photochemical quenching coefficient (qP). **(D)** Non-photochemical quenching coefficient (NPQ). **(E)** Sedoheptulose-1,7-bisphosphate esterase (SBPase). **(F)** Ribulose-1,5-bisphosphate carboxylase/oxygenase (Rubisco). **(G)** NADP-dependent glyceraldehyde-3-phosphate dehydrogenase (NADP-GAPDH). **(H)** Fluorescence kinetics (OJIP) curve. **(I)** Relative variable fluorescence Vt curve. **(J)** Differential kinetic ΔVt curve of relative variable fluorescence. The results presented the means ± SE of three independent experiments. The different lowercase letters above the bars indicate significant differences between treatments according to Duncan’s test (p < 0.05).

Chlorophyll fluorescence parameters are key indicators for studying plant stress responses. Compared to those in CK, the maximum photochemical efficiency (Fv/Fm) of tomato leaves in the HT group significantly decreased by 15.02%, whereas Fv/Fm in the HT+MT group increased by 11.66% relative to the HT group (**Figure 3A**). The actual photochemical efficiency [Y(II)] and photochemical quenching coefficient (qP) showed similar trends, both significantly decreasing under high-temperature stress. Conversely, the HT+MT treatment significantly increased Y(II) and qP by 31.44% and 71.92%, respectively, compared to those under HT conditions. Additionally, the NPQ in the HT group increased by 12.36% compared to that in CK, while NPQ in HT+MT decreased slightly relative to HT (**Figures 3B–D**).

SBPase, Rubisco, and NADP-GAPDH are essential enzymes in photosynthesis and play critical roles in the Calvin cycle. In the HT group, SBPase, Rubisco, and NADP-GAPDH activities decreased by 13.57%, 60.11%, and 27.13%, respectively, compared to those in CK. Conversely, SBPase, Rubisco, and NADP-GAPDH activities in the HT+MT group increased significantly by 8.48%, 124.39%, and 17.4%, respectively, compared to those in the HT group (**Figures 3E–G**).

The effects of melatonin on chlorophyll fluorescence kinetics (OJIP curve) and JIP-test parameters in tomato seedlings under high-temperature stress were determined. Compared to that of the CK group at room temperature, the OJIP curve of the HT treatment group showed significant changes, including decreased fluorescence intensity at J, I, and P points; a reduced J–P segment amplitude; and a more gradual curve. Melatonin application significantly alleviated changes in the OJIP curve under high-temperature stress, with increased fluorescence intensities at the O, J, I, and P phases. This suggests that exogenous melatonin mitigates the inhibition of photosynthetic electron transfer from QA to QB in tomato seedlings under high-temperature stress (**Figure 3H**). ΔK and ΔJ reflect ΔVt values at 0.3 ms and 2 ms, respectively, with **Figures 2I** and 3J based on Vt and ΔVt. As shown in the diagram, compared to those in CK, K and J phases appeared in the HT treatment, with ΔK and ΔJ values increasing to greater than 0. This indicates that high-temperature stress disrupted the oxygen-evolving complex (OEC), reducing electron transfer efficiency across plastoquinone (QA) and leading to a significant Q–A accumulation. Consequently, both the donor and acceptor sides of photosystem II (PSII) were severely damaged and inhibited. Compared to HT treatment, HT+MT significantly reduced ΔK values under high-temperature stress, indicating that melatonin alleviates OEC damage (**Figures 3I, J**).

The effect of melatonin on JIP-test parameters in tomato seedlings under high-temperature stress was determined. Compared to CK, HT treatment significantly increased Vj, Vi, and dV/dto and decreased ΨEo and PI_ABS_. Following HT+MT treatment, Vi and dV/dto significantly decreased, ΨEo increased, and Vj showed a slight decrease. The effects of melatonin on energy distribution per unit area in tomato seedlings under high-temperature stress showed that after HT+MT treatment, ABS/RC, DIo/RC, and ETo/RC increased by 21.66%, 54.27%, and 54.87%, respectively, compared to those in HT. However, the increase in TRo/RC was minimal. The impact of MT on specific activity parameters in tomato seedlings under high-temperature stress was analyzed. Compared to those in CK, the parameters ABS/CSm, ETo/CSm, and TRo/CSm decreased significantly under high-temperature stress by 30.58%, 57.51%, and 41.64%, respectively. DIo/CSm increased by 28.11% under high-temperature stress. ABS/CSm, DIo/CSm, ETo/CSm, and TRo/CSm increased significantly following exogenous melatonin application; compared to those in HT treatment, these parameters increased by 11.45%, 17.40%, 29.37%, and 8.99%, respectively (**Table 5**).

**Table 5:** Impact of exogenous melatonin on JIP-test, energy distribution per unit area, and specific activity parameters in tomato seedlings under high-temperature stress.

Treatments Parameters	CK	MT	HT	HT+MT
ΨEo	0.522 ± 0.007a	0.505 ± 0.003a	0.296 ± 0.039c	0.437 ± 0.009b
Vj	0.386 ± 0.004b	0.386 ± 0.007b	0.475 ± 0.07a	0.435 ± 0.009a
Vi	0.727 ± 0.007bc	0.718 ± 0.008c	0.81 ± 0.016a	0.777 ± 0.027ab
dV/dto	0.705 ± 0.006b	0.716 ± 0.013b	1.06 ± 0.126a	0.771 ± 0.046b
PI_ABS_	3.441 ± 0.252a	3.466 ± 0.245a	1.559 ± 0.597b	1.617 ± 0.148b
ABS/RC	3.215 ± 0.004a	3.162 ± 0.007a	2.396 ± 0.171c	2.915 ± 0.159b
DIo/RC	0.33 ± 0.005c	0.328 ± 0.004c	0.409 ± 0.035b	0.631 ± 0.032a
ETo/RC	1.357 ± 0.009a	1.34 ± 0.015a	0.738 ± 0.141b	1.143 ± 0.071a
TRo/RC	1.885 ± 0.003b	1.856 ± 0.013b	2.044 ± 0.018a	2.141 ± 0.057a
ABS/CSm	3,793.33 ± 29.80a	3,629.33 ± 37.90a	2,633 ± 26.41c	2,934.67 ± 34.26b
DIo/CSm	601 ± 7.42c	542.67 ± 11.02c	770 ± 8.30b	904 ± 14.88a
ETo/CSm	2,003 ± 24.17a	1,901.67 ± 28.85a	851 ± 25.10c	1,101 ± 22.06b
TRo/CSm	3,192.33 ± 69.31a	3,086.67 ± 29.67a	1,863 ± 44.71c	2,030.67 ± 42.52b

Different lowercase letters within the same column signify statistically significant differences between treatments (p < 0.05).

CK, control; MT, melatonin; HT, high temperature.

### 3.4 Regulation of the chlorophyll synthesis pathway by exogenous melatonin in tomato seedlings under heat stress

**Figure 4:**
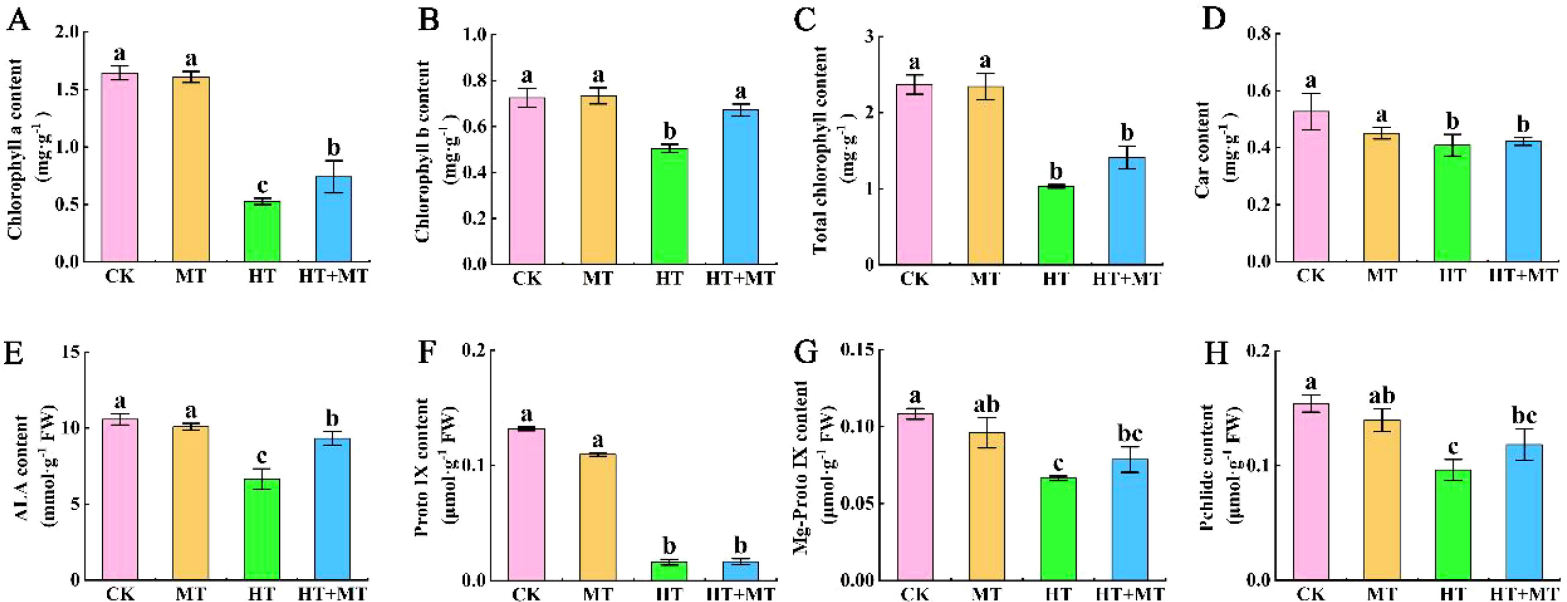
Effects of exogenous melatonin on chlorophyll content and chlorophyll synthesis precursor content in tomato seedlings under high-temperature stress. **(A)** Chlorophyll *a* content. **(B)** Chlorophyll *b* content. **(C)** Total chlorophyll content. **(D)** Carrot content. **(E)** 5-Aminolevulinic acid (ALA) content. **(F)** Protoporphyrin IX (Proto IX) content. **(G)** Mg-protoporphyrin IX (Mg Proto IX) content. **(H)** Protochlorophyllide (Pchlide) content. The results presented the means ± SE of three independent experiments. The different lowercase letters above the bars indicate significant differences between treatments according to Duncan’s test (p < 0.05).

Chlorophyll, a crucial photosynthetic pigment, plays a vital role in the photosynthetic system. Under HT stress, chlorophyll *a*, chlorophyll *b*, carotenoids, and total chlorophyll contents decreased significantly by 67.93%, 30.37%, 22.42%, and 56.43%, respectively, compared to those in CK. In the HT+MT treatment group, chlorophyll *a*, chlorophyll *b*, and total chlorophyll contents increased significantly by 40.51%, 33.09%, and 36.88%, respectively, compared to those in HT, while carotenoid content remained unchanged. MT application alleviates high-temperature stress damage to photosynthetic pigments, helping maintain chlorophyll balance (**Figures 4A–D**).

Glutamic acid, a precursor in chlorophyll synthesis, promotes production through several enzymatic reactions. 5-Aminolevulinic acid (ALA), protoporphyrin IX (Proto IX), Mg-protoporphyrin IX (Mg Proto IX), and protochlorophyllide (Pchlide) are essential precursors in the chlorophyll synthesis pathway. High-temperature stress significantly reduced ALA, Proto IX, Mg Proto IX, and Pchlide levels. Compared to HT, the HT+MT treatment significantly increased ALA, Mg Proto IX, and Pchlide levels by 40.15%, 18.37%, and 22.89%, respectively. These results indicate that melatonin effectively mitigates chlorophyll degradation under high-temperature stress (**Figures 4E, G, H**). Proto IX showed no significant change (**Figure 4F**).

### 3.5 Modulation of chlorophyll synthesis and degradation gene expression by exogenous melatonin in tomato seedlings under heat stress

**Figure 5:**
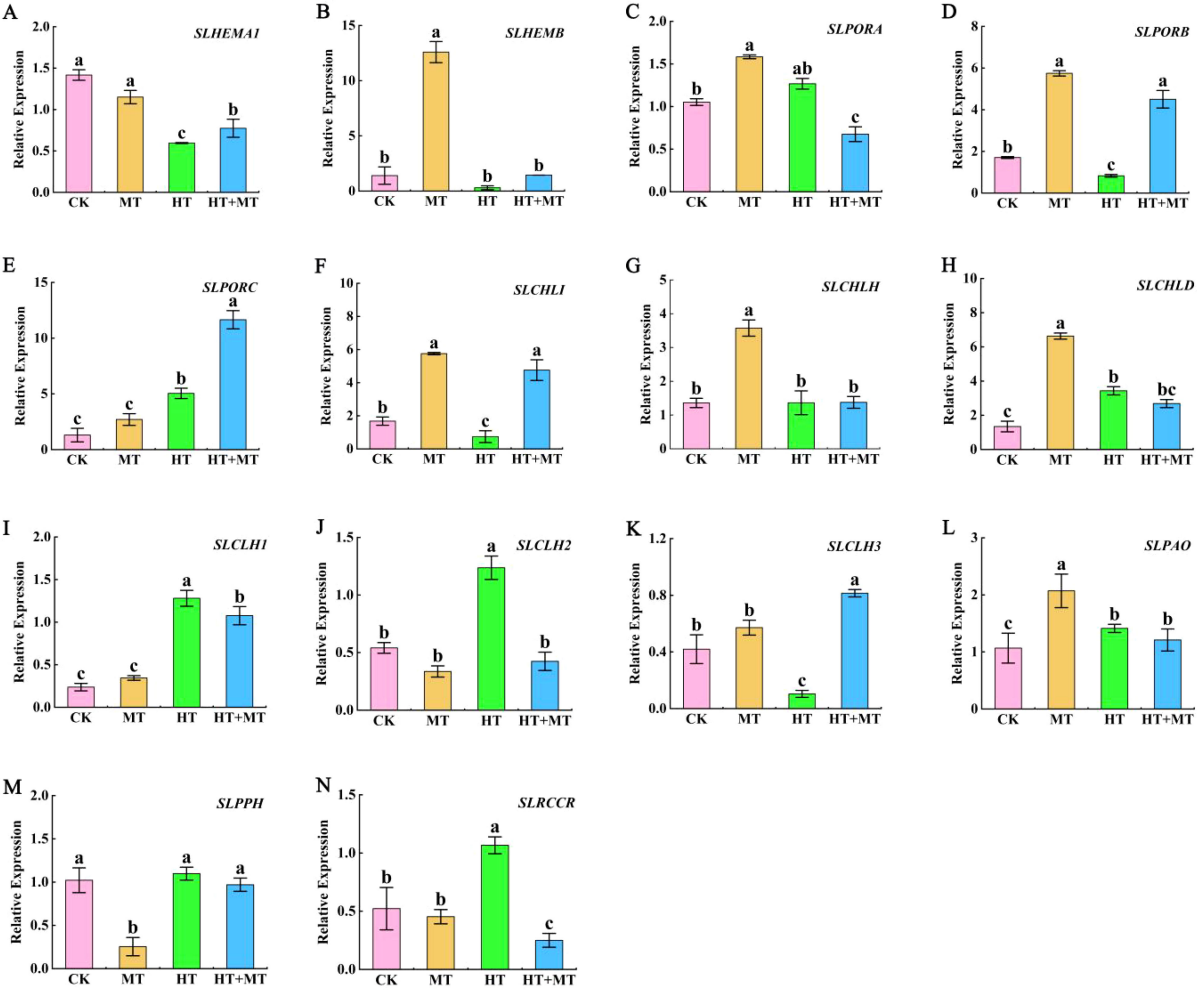
Expression analysis of chlorophyll synthesis and decomposition genes in tomato seedlings under high-temperature stress by exogenous melatonin. Chlorophyll synthesis gene. **(A)** *SlHEMA1*. **(B)** *SlHEMB*. **(C)** *SlPORA*. **(D)** *SlPORB*. **(E)** *SlPORC*. **(F)** *SlCHLI*. **(G)** *SlCHLH*. **(H)** *SlCHLD*. Chlorophyll catabolic gene. **(I)** *SlCLH1*. **(J)** *SlCLH2*. **(K)** *SlCLH3*. **(L)** *SlPAO*. **(M)** *SlPPH*. **(N)** *SlRCCR*. The results presented the means ± SE of three independent experiments. The different lowercase letters above the bars indicate significant differences between treatments according to Duncan’s test (p < 0.05).

To investigate how melatonin influences chlorophyll synthesis, we analyzed key genes from three essential gene families: *HEM*, *POR*, and *CHL*. In the *HEM* family, *HEMA1* is a key gene regulating the synthesis of ALA. The results showed that high-temperature treatment significantly decreased *SlHEMA1* expression, while HT+MT treatment increased its expression (**Figure 5A**). *SlHEMB* gene expression was reduced under high-temperature stress (**Figure 5B**). Following HT+MT treatment, *SlHEMB* expression showed no statistically significant increase. This suggests that *SlHEMA1* plays a key role in the chlorophyll synthesis pathway under melatonin-regulated high-temperature stress. In the *POR* family, HT treatment significantly increased *SlPORA* and *SlPORC* expression but reduced *SlPORB* expression. After HT+MT treatment, *SlPORB* and *SlPORC* expression levels were significantly upregulated (**Figures 5C–E**). In the *CHL* family, HT treatment significantly downregulated *SlCHLI* and upregulated *SlCHLD* expression compared to CK, with no change in *SlCHLH*. Compared to those in HT, *SlCHLI* expression was significantly upregulated in the HT+MT group, while *SlCHLH* and *SlCHLD* showed no significant change (**Figures 5F–H**).

The effects of MT on chlorophyll decomposition genes *CLH*s, *PAO*, *PPH*, and *RCCR* in tomato seedlings under high-temperature stress were determined. Under HT stress, *SlCLH1* and *SlCLH2* expression levels increased compared to those in CK. In the HT+MT group, *SlCLH1* and *SlCLH2* expression levels significantly decreased relative to those in the HT group. However, *SlCLH3* showed an opposite trend (**Figures 5I–K**). Compared to CK, HT treatment increased *SlPPH*, *SlPAO*, and *SlRCCR* expression. In the HT+MT group, *SlPPH*, *SlPAO*, and *SlRCCR* expression levels were downregulated compared to those in HT. This suggests that MT application at elevated temperatures may inhibit *SlCLH1*, *SlCLH2*, *SlPPH*, *SlPAO*, and *SlRCCR* expression, consequently suppressing chlorophyll degradation (**Figures 5L–N**).

### 3.6 The effect of exogenous melatonin on endogenous melatonin levels and the expression of related genes in tomato seedlings under heat stress

**Figure 6:**
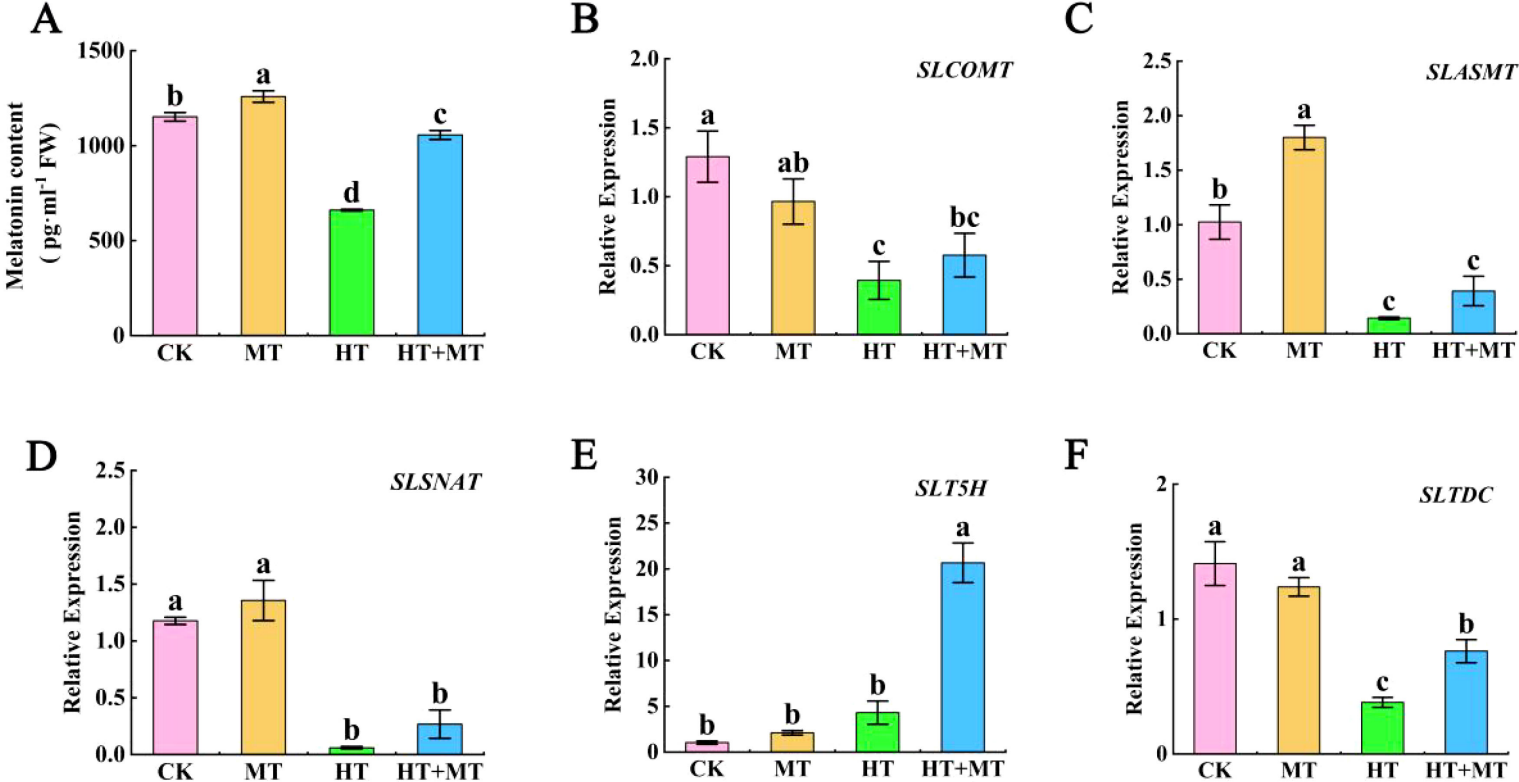
The effect of exogenous melatonin on endogenous melatonin levels and the expression of related genes in tomato seedlings under heat stress. **(A)** Melatonin content. **(B)** *SlCOMT*. **(C)** *SlASMT*. **(D)** *SlSNAT*. **(E)** *SlT5H*. **(F)** *SlTDC*. The results presented the means ± SE of three independent experiments. The different lowercase letters above the bars indicate significant differences between treatments according to Duncan’s test (p < 0.05).

At room temperature, the endogenous melatonin content in tomato seedlings treated with exogenous melatonin increased by 9.27% compared to that in untreated plants. In contrast, under high-temperature stress, the endogenous melatonin content in the CK group was significantly reduced by 42.68%. However, in the HT+MT treatment group, the endogenous melatonin content increased by 59.97% compared to that in the HT treatment group (**Figure 6A**). Under high-temperature stress, the expression levels of the *SlCOMT*, *SlASMT*, and *SlSNAT* genes were significantly downregulated compared to those in the CK. Notably, their expression levels increased in the HT+MT treatment group compared to the HT treatment group (**Figures 6B–D**). Additionally, the expression of the *SlT5H* gene increased under high-temperature stress compared to the CK treatment group, and this increase was further amplified after melatonin pretreatment. In contrast, the expression of the *SlTDC* gene was significantly reduced in the HT treatment group compared to the control group but significantly increased in the HT+MT treatment group following melatonin pretreatment under high-temperature stress. This indicates that exogenous melatonin is involved in endogenous melatonin biosynthesis, thereby alleviating heat-induced damage in tomato seedlings (**Figures 6E, F**).

### 3.7 The effect of exogenous melatonin on endogenous abscisic acid, auxin, and ethylene synthesis genes

**Figure 7:**
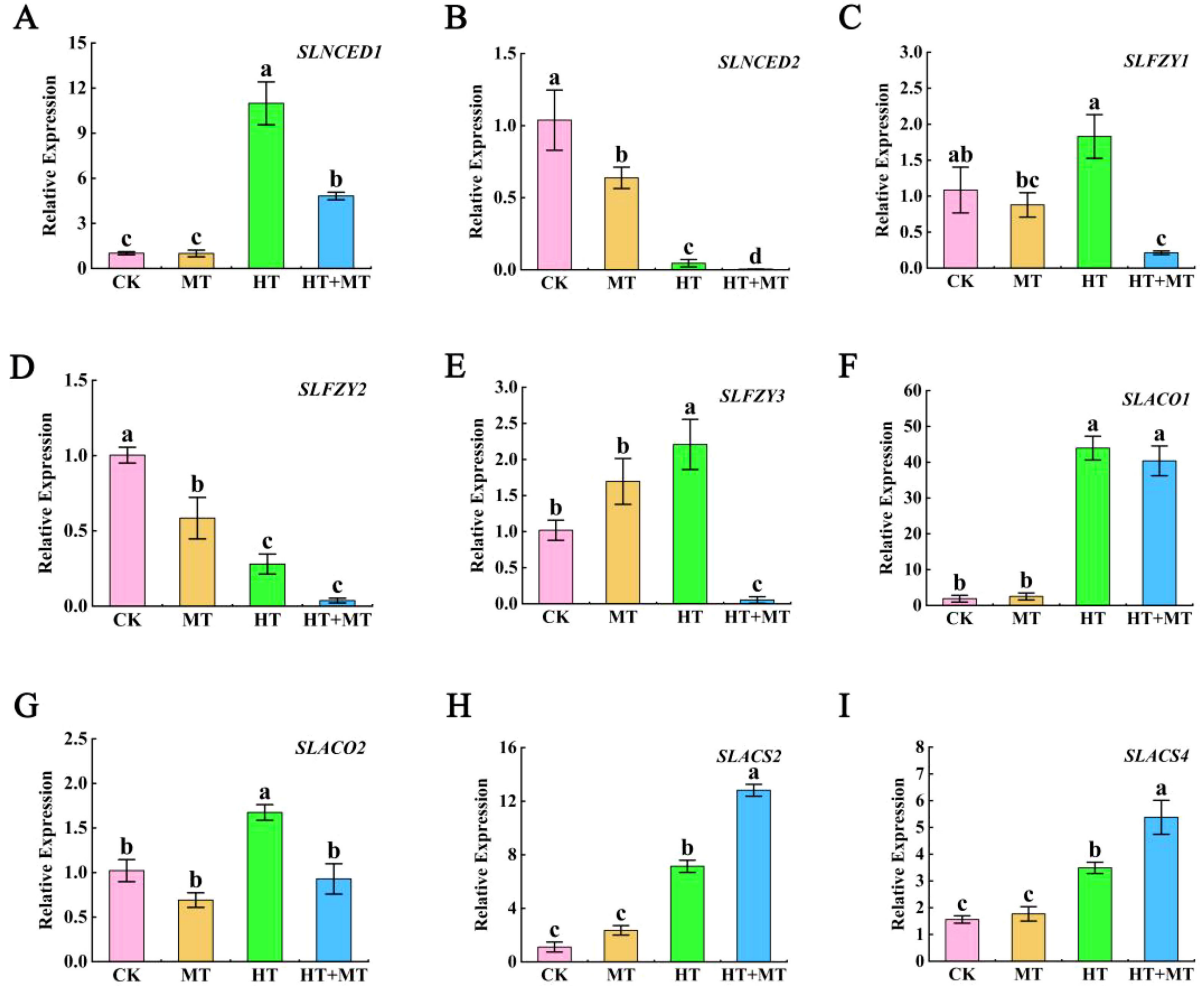
The effect of exogenous melatonin on endogenous abscisic acid, auxin, and ethylene synthesis genes. **(A)** *SlNCED1*. **(B)** *SlNCED2*. **(C)** *SlFZY1*. **(D)** *SlFZY2*. **(E)** *SlFZY3*. **(F)** *SlACO1*. **(G)** *SlACO2*. **(H)** *SlACS2*. **(I)** *SlACS4*. The results presented the means ± SE of three independent experiments. The different lowercase letters above the bars indicate significant differences between treatments according to Duncan’s test (p < 0.05).

As shown in the figure, under HT stress, the expression of the ABA biosynthesis gene *SlNCED1* was significantly upregulated compared to that in the CK. However, in the HT+MT treatment group, *SlNCED1* expression decreased compared to that in HT treatment alone. In contrast, the expression pattern of *SlNCED2* was the opposite of that observed for *SlNCED1* (**Figures 7A, B**). Under HT stress, the expression levels of the IAA biosynthesis genes *SlFZY1* and *SlFZY3* were significantly upregulated compared to those in the CK. However, when melatonin was applied under HT stress, the expression levels of *SlFZY1* and *SlFZY3* were significantly downregulated compared to those in HT treatment alone. In contrast, the expression of *SlFZY2* was significantly lower under HT stress compared to CK, and its expression was further reduced following melatonin application under HT conditions (**Figures 7C–E**). *SlACO1* and *SlACO2* are key regulatory genes encoding rate-limiting enzymes in the ethylene biosynthesis pathway. As shown in **Figures 7F**, **G**, their expression levels were significantly upregulated under HT stress compared to those in the CK. However, in the HT+MT treatment group, the expression levels of *SlACO1* and *SlACO2* were reduced compared to those in HT treatment alone, with *SlACO2* showing a particularly pronounced decrease (**Figures 7F, G**). *SlACS2* and *SlACS4* are members of the 1-aminocyclopropane-1-carboxylate synthase (ACS) gene family, which encode key rate-limiting enzymes in the ethylene biosynthesis pathway. Under HT stress, the expression levels of *SlACS2* and *SlACS4* were significantly upregulated compared to those in the CK. Moreover, their expression levels were further enhanced following HT+MT treatment (**Figures 7H, I**).

## 4 Discussion

High-temperature stress poses a significant threat to plant growth and agricultural sustainability. High-temperature stress severely impacts tomato growth and development, significantly reducing yield and quality (Mahajan and Tuteja, 2005). This study found that high temperatures significantly hinder normal tomato leaf growth. High-temperature stress causes new leaves to curl and droop and older leaves to yellow and wither, leading to significantly inhibited growth. Elevated temperatures disrupt the balance of ROS production and removal in tomato leaves, increasing levels of hydrogen peroxide, superoxide anions, and malondialdehyde. SOD and POD enhance the plant’s antioxidant defense system, improving antioxidant capacity to better cope with high-temperature stress (Haldimann and Feller, 2004). This study found that high temperatures increase CAT and APX activities. The experiment suggests that 100 μmol·L^−1^ MT effectively mitigates high-temperature stress in tomato seedlings, enhancing biomass, antioxidant enzyme activity, and proline and soluble sugar levels. MT treatment also reduces conductivity, malondialdehyde, hydrogen peroxide, and superoxide anion levels. These findings indicate that MT enhances tomato seedling resilience by stabilizing membranes, enhancing osmotic regulation, and increasing antioxidant activity, consistent with previous research on wheat seedlings under similar stress (Feng et al., 2007).

Carbon assimilation is a critical photosynthetic process, converting inorganic carbon dioxide into organic compounds through enzymatic reactions (Alissandratos and Easton, 2015). Stress conditions like drought, high temperatures, low temperatures, and salt stress can alter enzyme structures and damage active sites (Henriques, 2009). Rubisco is a bifunctional enzyme. High-temperature stress above 35°C inhibits Rubisco’s initial carboxylation activity in *Quercus pubescens* leaves, reducing the overall photosynthetic rate (Ouzounidou et al., 1997). SBPase is a crucial enzyme in the Calvin cycle (Raines, 2003). Studies have shown that SBPase overexpression enhances photosynthesis in transgenic rice under high-temperature stress, improving high-temperature resistance. This suggests that increasing SBPase to supply Rubisco with RuBP enhances plant resistance (Bilger et al., 1995). In this experiment, high-temperature stress significantly reduced SBPase and Rubisco activities. Under high-temperature stress, MT enhances SBPase and Rubisco activities, promoting carbon fixation and photosynthesis. Enhanced Rubisco and SBPase activities increase net photosynthetic rates and improve carbon assimilation capacity. NADP-GAPDH catalyzes the reduction of NADP+ to NADPH, supplying NADPH and ATP for reactions like carbon fixation (Zhang G. et al., 2014). Experimental results showed that MT application significantly increased NADP-GAPDH activity under heat stress, boosting NADPH production to support CO_2_ fixation and organic synthesis. These findings suggest that MT treatment alleviates high-temperature stress effects on the Calvin cycle by regulating key enzyme activity, thereby enhancing photosynthesis.

Chlorophyll fluorescence is an effective probe for assessing the actual state of plant photosynthesis (Hou et al., 2016). Fluorescence kinetic parameters reveal the absorption, transformation, transmission, and distribution of light energy in plants (Shao et al., 2007). Measuring chlorophyll fluorescence parameters allows for assessing photosynthetic efficiency and the physiological state of plants. Parameters like maximum fluorescence yield (Fm) and variable fluorescence (Fv) relate to PSII electron transfer efficiency, while quantum yield reflects the efficiency of converting light energy to chemical energy in photosynthesis (Zhang H. et al., 2018). This study found that high-temperature stress significantly reduced Fv/Fm and Y(II) values in tomato seedling leaves, causing PSII reaction center damage and obstructed electron transport. The decrease in Fv/Fm indicates plant stress, consistent with Zhang Guoxian’s findings (Wassie et al., 2020). Qp, the photochemical quenching coefficient, reflects the openness and efficiency of photosystem II in photosynthesis (Havaux et al., 1991).

This study suggests that high-temperature stress markedly diminishes the quantum yield of photochemical efficiency (qP). This reduction indicates impeded electron transport from the oxidation side to the PSII reaction center, reducing photochemical electron transfer efficiency and light energy utilization. Consequently, energy allocated for photochemical reactions is diminished, inhibiting the photochemical process. This aligns with Shao Ling’s research on *Arabidopsis thaliana*, with increased NPQ further supporting this conclusion (Zhang J. et al., 2014). This study found that MT application significantly enhanced Fv/Fm, Y(II), and qP while reducing NPQ in high-temperature-stressed tomato seedlings. This indicates that MT alleviates photoinhibition from high-temperature stress, enhancing light energy conversion efficiency and photosynthetic electron transfer capacity. MT effectively mitigates high-temperature stress damage to the photosynthetic mechanism of tomato seedlings, preserving PSII function.

Rapid chlorophyll fluorescence reflects the physiological state of plants. Measuring the intensity and kinetic curve of rapid chlorophyll fluorescence reveals the photosynthetic efficiency of tomato seedlings under high-temperature stress. The OJIP curve provides detailed information about PSII photosynthetic electron transport (Havaux and Tardy, 1999). This study found that high-temperature stress significantly altered the OJIP curves of tomato leaves, reducing the O, J, I, and P phases. MT restored the OJIP curve under high-temperature stress. The JIP-test quantitatively analyzes OJIP curve changes, reflecting light energy absorption, PSII reaction center activity on the acceptor and donor sides, and dynamic redox changes in electron transfer entities (Li et al., 2023). High-temperature stress significantly increased Vi and dV/dto, indicating reduced PQ electron-acceptance capacity. MT effectively reduced both Vi and dV/dto under high-temperature conditions. ΨEo represents the efficiency of energy absorption by the antenna and transfer to QB. MT application under high-temperature stress significantly increased ΨEo. This indicates that high-temperature stress hinders electron transfer from QA to QB in tomato leaves, reducing energy transfer efficiency, while MT alleviates this damage. PI_ABS_, the performance index for light energy absorption in photosynthesis, is highly sensitive to stress (Tanaka and Tanaka, 2006). This experiment showed that MT application increased PI_ABS_ in tomato seedling leaves, indicating enhanced photosynthetic capacity under high-temperature stress. Energy distribution changes in a single active reaction center in tomato leaves under different treatments were analyzed. High temperatures decrease light energy absorbed per unit reaction center (ABS/RC) and increase energy dissipated per unit reaction center (DIo/RC), indicating enhanced antenna absorption due to RC inactivation (Mathur et al., 2013). Variations in energy allocation per unit cross-sectional area of tomato leaves under different treatments were further analyzed. The results indicated that MT application at elevated temperatures enhanced electron transfer yield per unit area (ETo/CSm). MT under high-temperature stress protects PSII reaction center activity in tomato leaves by increasing absorption, capture, and electron transfer energy while reducing dissipated energy. MT treatment increased ABS/RC and TRO/RC ratios under high-temperature stress, indicating reduced light energy dissipation and increased energy capture for electron transfer. This improved the photochemical efficiency of tomato seedlings under high-temperature stress, protecting photosynthesis. By measuring the endogenous melatonin content and the expression levels of melatonin biosynthesis-related genes in tomato seedlings under high-temperature stress, it was observed that exogenous melatonin application during heat stress increased both endogenous melatonin levels and the expression of melatonin biosynthesis genes. These increases contribute to the regulation of osmotic adjustment substances, the scavenging of excessive ROS generated under high-temperature stress, and the protection of chloroplast structure and function, thereby maintaining photosynthetic efficiency (Ahammed et al., 2019; Jia et al., 2020).

Melatonin can interact with other phytohormones and signaling molecules, playing a crucial role in plant stress resistance. Studies have shown that under stress conditions, melatonin exhibits antagonistic or synergistic effects with other hormones throughout the physiological changes in plants (Kanwar et al., 2018). ABA, as a plant hormone, plays an indispensable role in the lifecycle of tomato seedlings. Studies have shown that under high-temperature stress, the ABA synthesis genes *NCED1* and *NCED9* in tomato thermo-dormant seeds are significantly upregulated (Geshnizjani et al., 2018). In this study, high temperature significantly increased the expression of the abscisic acid synthesis gene *SlNCED1*, inducing leaf senescence in tomato seedlings. Under heat stress, exogenous application of melatonin significantly downregulated the expression of the *SlNCED1* gene. Similar findings were reported by Mohammad Shah Jahan et al. in their study on tomato heat tolerance mediated by ABA and GAs (Ding et al., 2022). However, the expression of *SlNCED2* was contrary to their findings, possibly because *SlNCED1* is the key gene responsible for ABA biosynthesis in tomatoes under heat stress. IAA acts as a signaling molecule under drought and other stress conditions, regulating stomatal movement by causing guard cells to lose water, leading to stomatal closure. This process reduces water loss and enhances plant drought tolerance (Sarwat and Tuteja, 2017). Studies have found that melatonin signaling and auxin biosynthesis exhibit antagonistic interactions. In *Arabidopsis*, melatonin overexpression significantly downregulates the expression of key auxin biosynthesis genes *YUC1*, *YUC2*, and *YUC5* (Wang Q. et al., 2016). This study found that the gene expression of *SlFZY1* and *SlFZY3* was significantly upregulated under high-temperature stress in the *FYZ* family of *YUC* homologous genes in tomatoes, but *SlFZY2* was significantly downregulated. The expression of melatonin *SlFZY1* and *SlFZY3* was significantly downregulated when sprayed at high temperatures, indicating that melatonin has a negative regulatory effect on auxin synthesis genes under high-temperature stress. Studies have shown that under heat stress, the expression of ethylene biosynthesis-related genes *ACO1*, *ACO4*, *EREB*, and *ETR4* is significantly upregulated, which in turn induces the transcription of HSP genes and enhances the tomato plant’s response to high-temperature stress (Pan et al., 2019). ACS is a key rate-limiting enzyme in the ethylene biosynthesis pathway. ACS2 and ACS4 catalyze the conversion of *S*-adenosyl methionine (SAM) into 1-aminocyclopropane-1-carboxylic acid (ACC) (Khan et al., 2024). ACO1 and ACO2 catalyze the conversion of ACC into ethylene. In this study, it was found that under heat stress, the expression of the *SlACS2* and *SlACS4* genes was significantly upregulated. Exogenous application of melatonin further upregulated the expression of SlACS2 and *SlACS4* under heat stress. Additionally, in tomato seedlings under heat stress, the expression of *SlACO1* and *SlACO2* was significantly upregulated. However, the expression changes of *SlACO1* and SlACO2 upon exogenous melatonin application under heat stress were not as pronounced. This suggests that *SlACS2* and *SlACS4* are key genes involved in ethylene synthesis during melatonin application under heat stress. It indicates that melatonin and ethylene have a synergistic effect on plant growth, development, and stress response, jointly regulating physiological processes and enhancing plant stress tolerance (Sharma et al., 2023).

Plants possess photosynthetic pigments, including chlorophyll *a*, chlorophyll *b*, and carotenoids, which are essential for capturing light energy. However, under high-temperature stress, the concentrations of these pigments decline, leading to reduced absorption and conversion of light energy. This ultimately leads to a reduction in the photosynthetic rate (Tripathy and Pattanayak, 2012). Melatonin application significantly reduced chlorophyll degradation in tomato seedling leaves under high temperatures, protecting plants by maintaining photosynthetic potential under stress. Chlorophyll *a* and *b* synthesis and degradation involve 15 steps and 27 genes, making them complex processes (Wu et al., 2018). Each step is crucial, ultimately influencing chlorophyll levels (Zhang Z. W. et al., 2018). Chlorophyll metabolism includes both synthesis and catabolism. Chlorophyll synthesis occurs in four stages: ALA, protochlorophyllide (Proto IX), chlorophyll *a*, and chlorophyll *b* synthesis (Akram and Ashraf, 2013). Each stage involves enzyme-catalyzed reactions, and the downregulation of genes encoding these enzymes can reduce enzyme activity, blocking catalytic reactions and reducing intermediates (Notarnicola et al., 2023). This study found that high-temperature stress significantly reduced chlorophyll precursors ALA, Proto IX, Mg-proto IX, and Pchlide. These findings indicate that high-temperature stress blocks chlorophyll synthesis in tomato seedlings. After MT application at elevated temperatures, levels of ALA, Mg-proto IX, and Pchlide significantly increased. This indicates that melatonin enhances chlorophyll synthesis at high temperatures by regulating chlorophyll precursors. Proto IX showed no significant change, suggesting that some may be converted to the heme branch. This aligns with research on ALA-sprayed cucumbers’ salt resistance (Wu et al., 2022). *SlHEMA1* is a key gene encoding glutamyl-tRNA reductase in the chlorophyll synthesis pathway (Zhang et al., 2015). The study revealed that high-temperature stress markedly decreased the expression of *SlHEMA1*. Similar results were observed under hydrothermal stress in rice (Dalal and Tripathy, 2012). In this experiment, the MT application significantly upregulated *SlHEMA1* expression under high-temperature stress. This indicates that MT promotes endogenous ALA synthesis under high-temperature stress by upregulating *SlHEMA1* and enhancing Glu-tRNA reductase activity. The POR enzyme is essential for chlorophyll synthesis, facilitating the conversion of Pchlide to Chlide (Schoefs and Franck, 2003). POR has three isoenzymes—PORA, PORB, and PORC—encoded by the *PORA*, *PORB*, and *PORC* genes (Blomqvist et al., 2008). Reports have indicated that under heat stress, POR protein levels decrease with light exposure, whereas under cold stress, yellowing and POR protein levels in light-grown seedlings remain stable (Mohanty et al., 2006). In this study, *SlPORA* and *SlPORC* expression levels significantly increased at elevated temperatures. Following the MT application, *SlPORA* expression was downregulated under high-temperature stress, while *SlPORC* expression was upregulated. The magnesium chelating enzyme (MCH) consists of three subunits—CHLH, CHLI, and CHLD—with CHLH primarily responsible for catalytic activity (Masuda et al., 2003). Jin et al. reported that chlorophyll synthase (CHLG) and protochlorophyllide oxidoreductase (POR) expression increased in melon seedlings under salt–alkali stress (Jin et al., 2019). In this study, high-temperature treatment significantly reduced *SlCHLI* expression compared to the control, while MT application during stress significantly increased *SlCHLI* expression. These findings suggest that the *HEM*, *POR*, and *CLH* family genes are crucial for the chlorophyll synthesis pathway. Furthermore, MT modulates these gene expressions, counteracting high-temperature stress on chlorophyll synthesis and providing protection under elevated temperatures.

**Figure 8:**
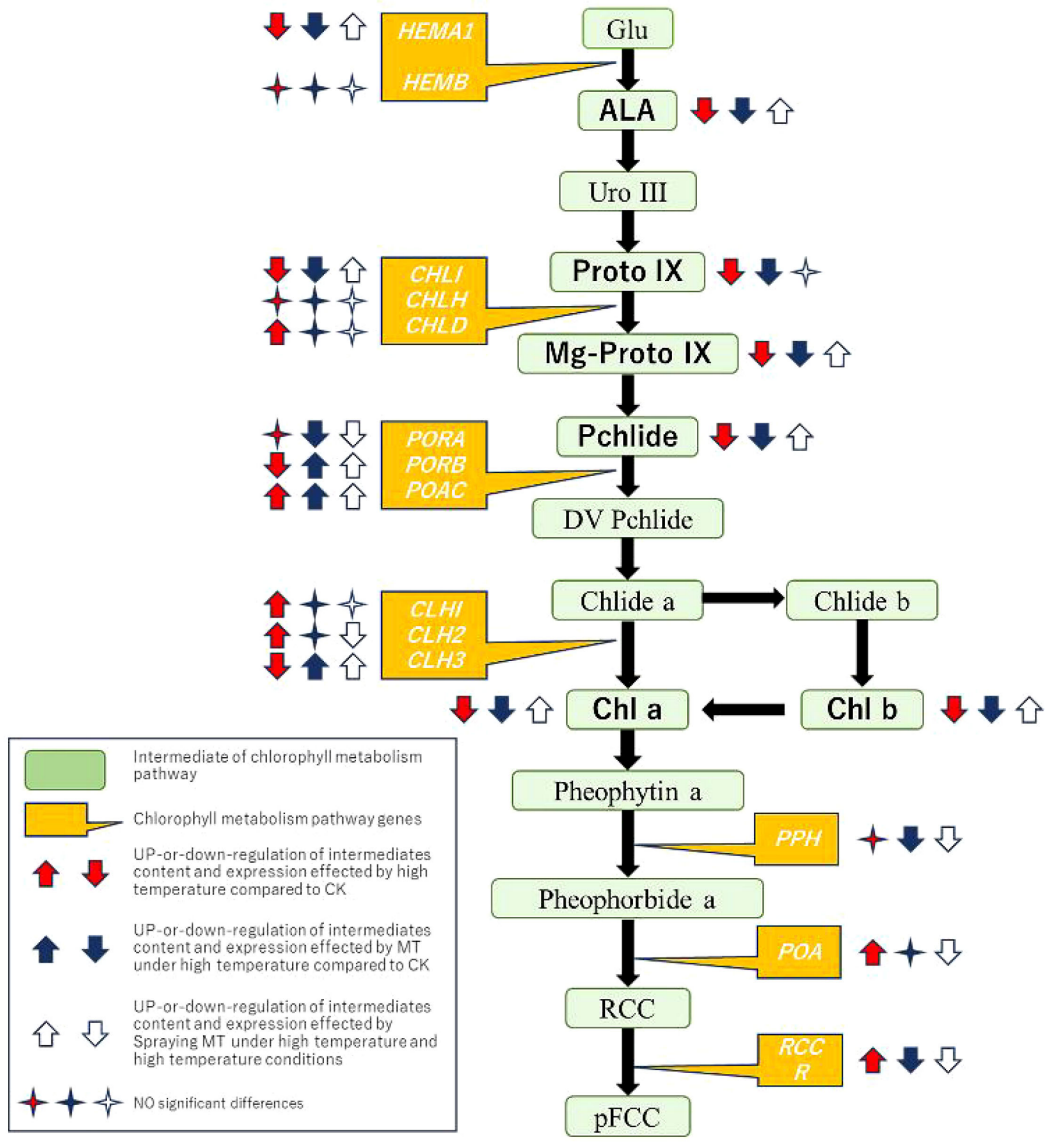
A model of exogenous melatonin participating in chlorophyll metabolism to induce tomato seedlings to resist high-temperature stress.

Chlorophyll degradation mechanisms vary across species and treatment conditions in green plant tissues (Hörtensteiner, 2006). Enzymes such as CLH, magnesium chelatase (SGR and SGRL), PAO, and RCCR play crucial roles in chlorophyll degradation (Sakuraba et al., 2014). This experiment measured expression levels of the *CLH*, *PAO*, *PPH*, and *RCCR* genes. *SlCLH1*, *SlCLH2*, and *SlCLH3* are key genes encoding the chlorophyll-degrading enzyme chlorophyllase (CLHase). The primary function of CLHase is converting chlorophyll *a* to deglycosylated chlorophyll *a*. This study found that high-temperature conditions significantly upregulated *SlCLH1* and *SlCLH2* expression. Low-temperature treatment in Japanese knotweed significantly increased *ZjCLHase*, *ZjPAO*, and *ZjRCCR* expression, accelerating chlorophyll degradation (Zhang et al., 2022). PPH is the key enzyme catalyzing the conversion of pheophytin a to Pheide a. PAO catalyzes the conversion of pheophytin to red chlorophyll catabolites (RCC). RCCR is the final enzyme in chlorophyll degradation, catalyzing the conversion of RCC to the colorless, blue-fluorescent product pFCC (Luo et al., 2022). This study demonstrated that high-temperature stress significantly upregulated *SlPAO*, *SlPPH*, and *SlRCCR* expression. This may affect enzyme activity and accelerate chlorophyll degradation (Tang et al., 2011). MT application significantly reduced *SlCLH1*, *SlCLH2*, *SlPPH*, *SlPAO*, and *SlRCCR* expression, effectively inhibiting chlorophyll degradation under high-temperature stress. In conclusion, melatonin enhances leaf adaptability to high temperatures, stabilizes PSII and PSI structure and function, and improves photochemical efficiency in tomato leaves under high-temperature stress by regulating key chlorophyll metabolism genes (**Figure 8**).

In summary, treatment with 100 μmol·L^−1^ MT significantly enhanced the growth of tomato seedlings under high-temperature stress. This treatment mitigated ROS damage, increased antioxidant enzyme activity, and improved membrane stability in the seedlings. MT also elevated the activity of key photosynthetic enzymes, specifically Rubisco and SBPase. MT reduced damage to PSII, facilitated electron transfer from QA to QB in the photosynthetic electron transport chain, and enhanced overall photosynthetic performance under high-temperature conditions. Additionally, MT protects photosynthesis by modulating chlorophyll metabolites and regulating key photosynthetic genes.

## Data Availability

The original contributions presented in the study are included in the article/**Supplementary Material**. Further inquiries can be directed to the corresponding authors.
