# Supplementary material for: Protective mechanisms of exogenous melatonin on chlorophyll metabolism and photosynthesis in tomato seedlings under heat stress
Source: Front Plant Sci. 2025 Feb 4;16:1519950. doi: 10.3389/fpls.2025.1519950 (PMC11833508; doi:10.3389/fpls.2025.1519950)
Supplement: Supplementary file 1 [file DataSheet1.pdf]

Supplementary table 1 Gene primer sequences used in fluorescence quantitative PCR

Gene	Gene full name	Accession number	Forward primer 5'-3'	Reverse primer 5'-3'
SlHEMA1	Glutamyl-tRNA Reductase 1	SGN-U579959	CAGTATCCACACTACGCCTG	CAGGATGGAGATCTATGTTGTG
SlHEMB	Glutamate-1-Semialdehyde 2,1-Aminomutase	SGN-U579023	GTAATCGTAGGTCGTCAGCA	TGGATGTTATAGGCTTGGATGGA
SlPORA	Protochlorophyllide Reductase A	Solyc12g013710	GCAATCACCCAGTCTACCTC	GGCTATAGGCGAGTCAGGAG
SlPORB	Protochlorophyllide Reductase B	Solyc10g006900	ACTAACCATCTTGGTCATTTCC	CAACAACAGGGCTATTCAGAG
SlPORC	Protochlorophyllide Reductase C	Solyc07g054210	CAACAACAGGGCTATTCAGAG	CTGAATCTGGAAAGAGACTTGC
SlCHLI	Magnesium Chelatase Subunit I	Solyc10g008740	GAATATCAATTTCACACCCTGCTC	CAGAACTAAGAGTGAAGATCGTC
SlCHLH	Magnesium Chelatase Subunit H	Solyc04g015750	AAGAAGGTGCCATTGTATCAG	GACTTGGAGAGTTTGGATGG
SlCHLD	Magnesium Chelatase Subunit D	Solyc04g015490	CCATTGACCGTGAGATAGGA	CTGAAGAGTGGGAAGATGGG
SlCLH1	Chlorophyllase 1	Solyc06g053980	GGTAGACTTGCTAGTGACCTG	CAAGCTGGCTTGCAACATTCG
SlCLH2	Chlorophyllase 2	Solyc09g065620	CTCTAAAATTCTCAGCACTCC	GACCATAATCCTTAGCAAGG
SlCLH3	Chlorophyllase 3	Solyc09g082600	CTCATGTTGGGCCAAATTTG	ACCATAAGTTGCCTTTCCTC
SlPAO	Pheophorbide a Oxygenase	Solyc11g066440	GCATTCCGAAATTGGCTTAGAC	GCTAATCCAGCACTTATAATTGC
SlPPH	Pheophytinase	Solyc01g088090	GTGTCGAATGAACAATGTACC	MCCATTGAGAAGTCATTGATCC
SlRCCR	Red Chlorophyll Catabolite Reductase	Solyc03g0444470	TTTCATACTTGGTAGTTGGGTTCA	GTCCTTTCGCGGAGGTAGAT
SlCOMT	Caffeic Acid O-Methyltransferase	Solyc03g080180.2	TGCTTAGGCTTCTTGCTGCTTACTC	ACGGAAACACCATCGGCATTCTTAG
SlASMT	Acetylserotonin O-Methyltransferase	Solyc12g009110.1	GGTTGTTGTTCCACTTCCCTCTA	TTGAACATAGCCGTTTTTGCC
SlSNAT	Serotonin N-Acetyltransferase	Solyc10g074910.1	CTCTTAATTGCTGCCGTTGTCAACC	TTCTTGCCAAACCCGGATCTGATG
SlT5H	Tryptamine 5-Hydroxylase	Solyc01g010430.2	AGCAAAAGCAGTAAGGCTAAGAAGT	CATCACGATCTTGAATCACTGTTTC
SlTDC	Tryptophan Decarboxylase	Solyc07g054860.1	CGCTTTATCTCCCGCAGTTCTACG	TGAGAGGATCGACGGCTGTAGTG
SlFZY1	Fuzzy 1	XM_004235308	TTTGATGGAGTCTGTGGGTTTTGG	TGGAATTTCTGGTATAACTGCCTCTG

SlFZY2	Fuzzy 2	XM_004235309	TCTGTGAACTACCACTTATGCCATTC	CGGTCATAACAAGCGGATACTACG
SlFZY3	Fuzzy 3	XM_004235310	CGAGATCAGCCCACGATTCAATG	TAGCCACCACAAGCCACCTAC
SlACO1	1-Aminocyclopropane-1-Carboxylate Oxidase 1	NM_001247095	TATTTATTCAATACACTTAGGAAAAC A	ACTTGAGAGATATTAGAAGTAGGAAGA
SlACO2	1-Aminocyclopropane-1-Carboxylate Oxidase 2	NM_001247096	AAATTTTGGGACTAAAGTAAGTAACT A	TAAATTTAGGGTAAACTTGTTTGTTAT
SlACS2	1-Aminocyclopropane-1-Carboxylate Synthase 2	NM_001247097	TATGGAGAGTTATTATAAACGATGTT A	CTAAGTACATAGACCAGTTGTCAATAC
SlACS4	1-Aminocyclopropane-1-Carboxylate Synthase 4	NM_001247098	ATTCACTAGAGGACTTGAAGAAATAG	CAAGCTTTATAACTTTATTTGATTGTA
SlNCED1	9-Cis-Epoxycarotenoid Dioxygenase 1	NM_001247099	ACGATGTGATTCAGAAGCCATACC	CAGGAATGACGACGAAGTTCTCAG
SlNCED2	9-Cis-Epoxycarotenoid Dioxygenase 2	NM_001247100	ACTGGAGTTGCTAATGCTGGATTG	AACTGTTTGAAGATCGCCAGAAGG
SlActin	Actin	Solyc04g011500	TGGGTCAAAAAGACGCCTATG	ATAATCTGGGTCATCTTTTCACGA
